# Medical education reform: a catalyst for strengthening the health system. *Are we ready, Canada?*

**Published:** 2019-11-28

**Authors:** Layli Sanaee

**Affiliations:** 1 Department of Medicine, Division of Emergency Medicine, University of Toronto, Ontario, Canada.; 2 University Health Network, Ontario, Canada.

Medical specialist education in Canada is transitioning from a time-based model to a competency-based one, called Competence by Design (CBD).^1^^,^^2^ The Royal College of Physicians and Surgeons of Canada (Royal College) launched CBD in July 2017, with the fundamental aim of aligning physician competencies with societal health needs.^1^^-^^3^ The term health systems is defined by the World Health Organizations (WHO) as “*all organizations, people and actions whose primary intent is to promote, restore or maintain health*.”^4^ Accordingly, medical education can be conceptualized as part of the health system, and CBD seen as a health system intervention. Given the dynamic nature of health systems, with multiple complex relationships between subsystems, an intervention seemingly limited to one element often has system-wide implications.^5^ Viewing CBD implementation through a systems lens can help develop parallel interventions and inform policy and management direction. By taking advantage of interconnected subsystems within the health system it is possible to mitigate unintended consequences while simultaneously identifying areas that can be strengthened.^5^^,^^6^

The following analysis applies CBD implementation as a case study, using the World Health Organization (WHO) Health System Framework for Action to illustrate potential implications and related opportunities of medical health education reform for health system functions, from my perspective.^4^ Frenk et al. (2009) provide a comprehensive overview of the transformational capacity of health care professional education reform on health systems.^6^ They offer an adaptation of the Health System Framework for use in conceptualizing the education system.^6^ In 2014, the Royal College published an in-depth report of recommendations for the reform of postgraduate medical education in Canada, going beyond curriculum to consider several aspects of the overall system.^2^

I hope to offer a unique reflective analysis of a specific reform feature in educating specialist medical doctors (referred to as residents), early in implementation and with a health systems perspective. The focus is on selected immediate and mid-term consequences of CBD in Canada. Not all health system implications or opportunities will be covered, nor will the education of general practitioners, who are overseen by a separate college.^7^ Given the close interactions between elements, some Building Blocks of the Framework will be discussed together, while discussions of Governance will be interspersed throughout.

## Competence by Design: Health system lens

***Health financing****Most* residents in outcome-based programs like CBD are expected to progress to independent practice earlier.^8^^,^^9^ Paradoxically, given that CBD is projected to better detect competency deficiencies, some learners may have a longer duration of training than in the current model.^9^^,^^10^ Regardless of the net effect, the length of time spent as a learner will not be as well-defined as before. Additionally, with an emphasis on diverse learning settings, CBD may result in greater movement of residents between hospitals, clinics, and even provinces.^2^ Annual variation has potential budgeting implications for faculties, hospitals, and ministries of health — all of which currently finance aspects of postgraduate medical education.^2^^,^^7^ This variation will make it necessary to seriously consider how best to adapt current financing models and resident salary negotiation procedures. This may include pooling funds among disciplines or academic centres, and greater coordination among stakeholders. Ultimately, CBD implementation brings opportunities to strengthen financing structures by reducing fragmentation and duplication and increasing transparency.^2^^,^^7^

CBD requires direct resident supervision and the formation of competence committees, which will be responsible for assessing eligibility to progress.^11^ Currently, there is significant variation among incentive and remuneration schemes for clinicians involved in teaching or serving on committees. Alongside CBD implementation, residency programs must deliberate as to whether committee members will be remunerated. As a result, calls to standardize teacher remuneration and incentives across the province or country may arise, particularly since a greater number of sites not affiliated with a university may supervise resident physicians. In the current economic climate, remuneration may be scaled back, which could have an effect on the supply of clinician teachers.

***Health workforce and service delivery*** Canadian academic hospitals rely heavily on specialist residents to staff clinics, operating rooms, medical wards, and overnight shifts.^2^^,^^7^ With a shorter duration of residency and more off-site rotations, academic hospitals may experience doctor shortages and a negative effect on patient care. If tasks now carried out by residents are shifted to attending physicians (such as in-house call shifts), some physicians may leave the academic setting. The additional direct supervision and workplace assessment requirements of CBD may have a similar consequence. With medical schools appropriately emphasizing the societal need for generalists rather than specialists, it is both anticipated and desired that fewer students will choose a specialist career.^3^ These effects could synergize with CBD implementation, creating doctor shortages and service delivery and clinical teaching impacts across academic health sciences centres.

One approach to both adapt to this potential consequence and strengthen the underlying processes is to design a more coordinated health human resource (HHR) management strategy across different strata — academic institutions, regionally, provincially and at the inter-professional level.^12^ Improved coordination can help resolve the current imbalanced proportions of medical school graduates to residency entrance spots; specialists to employment opportunities; specialists to generalists; and urban to rural physicians. As Maudsley et al. (2014) insightfully highlighted, if academic health science centres remain the principal regulator of the number of specialists trained, without regional or national coordination, the needs of academic institutions will continue to take priority.^12^ They additionally offered, “we need to move away from the notion that students and residents have an inalienable right to practise in the specialty and scope of their choosing without regard to societal need.”^12^ Although restrictions on resident positions have evolved since then, Maudsley et al. illuminate the importance of considering diverse perspectives in a balanced manner during regulatory decision- making. A coherent strategy would also better coordinate the human resource management (and training) of physicians with international medical graduates, nurse practitioners, registered nurses, physician assistants, and midwives.^2^^,^^7^ Such cohesion has great potential for ultimately positively affecting the health of individuals, communities, and populations.

Strengthening HHR management processes is difficult to put into operation. Several stakeholders are involved and students’ preferences cannot be entirely disregarded. The Royal College has emphasized HHR coordination in several submissions to the Federal Advisory Panel on Health Innovation and the House of Commons Standing Committee on Health.^13^ Perhaps coordination and strategic planning can begin within current structures, rather than being deferred until the government creates a new and separate agency.

***Information and technology*** Ideally, CBD implementation will be monitored closely and evaluated in real time. Choosing indicators to determine its impact and developing the capacity for measurement will prove challenging. Thus, opportunities to strengthen monitoring and interpretation of learner outcomes will be plentiful, as well as the links to patient and population outcomes. The Royal College already works closely with organizations such as the Canadian Institutes of Health Information to collect and analyze important data related to specialists. CBD implementation will push the frontiers of harmonization; for example, by streamlining the creation of “Health Intelligence Units”^14^ — a type of agency not yet in existence, but proposed as being charged with the surveillance and analysis of the health of a population and its corresponding human resources.^14^

***Governance and leadership*** It is clear that CBD implementation has several implications for governance and leadership. As explored above, this includes impacts on medical education financing models, HHR management, and the alignment of data collection and analysis. CBD implementation will test current structures, and likely propel adaptation. Organizations such as the Royal College may find they are taking on leadership roles and encouraging better coordination without being given a specific mandate or designation from the government. Through innovation, assertiveness, grassroots collaboration, and removal of duplication, CBD implementation can act as a catalyst for visionary leaders to create a stronger health system.

## Limitations and next steps

The effects of CBD implementation across all specialty programs in Canada are not yet known. This brief analysis is informed by a health systems conceptual framework, stakeholder perspectives, and a limited amount of experience; but primarily represents my opinions as the sole author. Such limitations are expected because of time lag and the nature of opinion-based analysis, but also reveal the importance of sophisticated evaluation, intentional consultations, and reflection on learning throughout CBD rollout. Many academic centres have experience with competency-based medical education, both internationally and within Canada due to staggered implementation. The orthopedic surgery residency program at the University of Toronto, for example, transitioned to a competency- based model in 2009, and have shared their learning beyond the direct impacts of curriculum.^10^^,^^15^ The College of Family Physicians of Canada began implementation of a competency-based curriculum in 2011.^16^^,^^17^ Reflections on learning from this diverse group, in particular regarding any experience with strengthening health systems functions, are invaluable for Canadian decision-makers and front- line clinicians alike. There are still questions regarding how to ensure widespread participation in the generation of such knowledge and its systematic dissemination and application throughout CBD rollout.

Some may view a health system lens as not necessary for CBD implementation, given it is relatively less complex than some other outlooks when considering the full spectrum of health system interventions.^5^ The comprehensiveness of this analysis counters that perspective, as using the Health System Framework systematically exposes interactions between subsystems.^3^^,^^5^ Perhaps the value in applying a health systems lens, then, is not to attempt to predict the outcomes of CBD implementation with a high degree of accuracy; but rather, to build capacity in analyzing systems-wide impacts of an intervention. Such capacity can in turn be used to unearth opportunities for strengthening health systems functions.

Going beyond identifying the system-wide implications and possibilities accompanying CBD implementation will prove essential. Next steps include consensus-based priority-setting and feasibility assessment by stakeholders, including community partners. Not every opportunity can or should be acted on, but each merits serious reflection. Given the broad scope of opportunities explored, several distinct but cohesive stakeholder deliberations should be considered. Specific representatives may be invited from: faculties of medicine, postgraduate medical education, regional public health authorities, registered nurse associations, ministries of health, hospital associations, patient advocacy groups, community organizations, the Royal College, the College of Family Physicians of Canada, and the Canadian Institutes of Health Information.

### Conclusions

Medical education reform in Canada is long overdue to ensure that physicians’ competencies will continue to adapt to the evolving health needs of society. This period of transition to a competency- based model provides a unique opportunity for parallel changes to strengthen the related health system processes.

Viewing CBD implementation through a health system lens has allowed for a systematic examination of its implications for Health System functions and relationships. I explored opportunities for strengthening the system, including restructuring the financing of medical education, designing a coherent HHR management strategy, standardizing teaching incentive schemes across geographical regions, and improving capacity for data collection and analysis. Given that stronger governance leads to greater efficiency and a more responsive system as a whole, it is not surprising that the majority of opportunities are closely linked with governance processes.

Since all reforms share the objective of improvement, we must not inadvertently create restrictions of scope. The commitment and coordination of a number of agencies is required to act on the potential that CBD brings. Using a Health Systems Framework at multiple stages of implementation will help. Returning to the question, Are we ready?, the answer is simple: we must become ready. It is time for action.
